# Use of Economic Compensation to Increase Demand for Voluntary Medical Male Circumcision in Kenya: Qualitative Interviews With Male Participants in a Randomized Controlled Trial and Their Partners

**DOI:** 10.1097/QAI.0000000000001047

**Published:** 2016-10-06

**Authors:** Emily Evens, Michele Lanham, Kate Murray, Samwel Rao, Kawango Agot, Eunice Omanga, Harsha Thirumurthy

**Affiliations:** * Health Services Research, FHI 360, Durham, NC;; † Social and Behavioral Health Sciences, FHI 360, Durham, NC;; ‡ Carolina Population Center, University of North Carolina at Chapel Hill, Chapel Hill, NC;; § Impact Research and Development Organization, Kisumu, Kenya; and; ‖ Department of Health Policy and Management, Gillings School of Global Public Health, University of North Carolina at Chapel Hill, Chapel Hill, NC.

**Keywords:** male circumcision, economic interventions, Kenya, demand creation, qualitative research

## Abstract

Interventions to increase demand for medical male circumcision are urgently needed in eastern and southern Africa. Following promising evidence that providing economic compensation can increase male circumcision uptake in Kenya, there is a need to understand the role of this intervention in individuals' decision-making regarding circumcision and explore perceptions of the intervention and concerns such as coercion.

**Methods:**

As part of a randomized controlled trial in Kenya that found compensation in the form of food vouchers worth US $8.75–US $15.00 to be effective in increasing male circumcision uptake, we conducted qualitative in-depth interviews with 45 circumcised and uncircumcised male participants and 19 female partners to explore how compensation provision influenced the decision to get circumcised. Interview transcripts were coded and an inductive thematic analysis was conducted to identify patterns in decision-making.

**Results:**

Interviews revealed that compensation promoted circumcision uptake by addressing a major barrier to male circumcision uptake: lost wages during and after the circumcision procedure. Participants who did not get circumcised perceived the compensation amounts to be insufficient for offsetting their costs associated with getting circumcised or reported having nonfinancial barriers that were not addressed by the intervention, such as fear of pain. Participants also reported that they did not feel compelled to get circumcised for financial gain. Female partners of circumcised participants felt that the intervention helped to motivate their partners to get circumcised.

**Conclusions:**

The results suggest that the provision of economic compensation is an acceptable intervention that can address an important barrier to male circumcision uptake. Providing compensation to circumcision clients in the form of food vouchers warrants further consideration in voluntary medical male circumcision demand creation efforts.

## INTRODUCTION

Male circumcision is an important component of combination HIV prevention in high prevalence regions of sub-Saharan Africa. Despite efforts to promote voluntary medical male circumcision (VMMC), however, the number performed has fallen short of targets in many countries, particularly among men aged over 25 years, who are at increased risk of acquiring HIV in many countries. There is a vital need for demand creation interventions that can address reported barriers to VMMC and increase circumcision prevalence.^1,2^

A commonly reported barrier to VMMC uptake among adult men is concern about lost wages during and after the procedure.^3–5^ Other reported barriers include fear of pain, concerns about the recommended 6-week period of sexual abstinence after circumcision,^6,7^ and men's concern that female partners may not approve.^3–5,8–10^ A qualitative study we conducted in rural areas of Kenya's Nyanza region identified financial concerns as the leading barrier among adult men.^5^ Many men expressed concern about providing for their families in the days after undergoing circumcision, and these were especially pronounced among married men. Study participants and local stakeholders identified a food voucher or cash transfer program as a priority intervention to increase VMMC uptake.

Building on the qualitative study and a strong theoretical and empirical rationale for using economic interventions to promote uptake of health interventions,^11,12^ we recently conducted a randomized controlled trial (RCT) in Kenya that found economic compensation to be effective in increasing VMMC uptake among 25- to 49-year-old men within a period of 2 months.^13^ Participants randomized to receive food vouchers worth 700 Kenya Shillings (KES) or 1200 KES (approximately US $8.75 and US $15.00, respectively) conditional on becoming circumcised were significantly more likely to get circumcised than participants randomized to receive a voucher for only the cost of transport (200 KES or US $2.50) or no compensation. In the US $8.75 and US $15.00 groups, VMMC uptake within 2 months was 6.6% and 9.0%, respectively, compared with 1.9% and 1.6% in the US $2.50 and the no compensation groups, respectively.

The promising RCT results give rise to important questions regarding why compensation provision motivated VMMC uptake for some men and, equally importantly, why it did *not* do so for other men. Moreover, despite evidence on the effectiveness of economic interventions and their widespread use in some countries,^12^ few studies identified whether such interventions succeed because they directly address economic barriers, if they coerce individuals to undertake an intervention out of need for the financial incentive, or if instead they “nudge” individuals toward desired health behaviors, as suggested by behavioral economics theories of present-biased decision-making.^11,14^ To complement the RCT and gain further insight on how compensation provision for VMMC was perceived, we conducted a qualitative study among trial participants and their female partners.

## METHODS

The study received ethical approval from the University of North Carolina at Chapel Hill and Kenyatta National Hospital/University of Nairobi. The study took place in the Nyanza region of western Kenya in collaboration with Impact Research and Development Organization, the primary provider of VMMC services in the study area.

Details and results for the RCT that this qualitative study was part of have been reported elsewhere.^13^ Trial participants met the following eligibility criteria: age between 25 and 49 years, self-reported to be uncircumcised, and no intention to leave the study area in next 3 months. Participants were randomized to a control group or 3 groups that were offered different amounts of food vouchers *conditional* on VMMC uptake within 2 months. The food voucher amounts were chosen because they approximated the costs of transportation from most participants' homes to a clinic or dispensary (US $2.50), the costs of transportation plus 1–2 days wages for most men (US $8.75), and the costs of transportation plus 3–4 days wages for most men (US $15.00). Importantly, the largest food voucher amount (US $15.00) was comparable to but not in excess of the likely opportunity costs associated with circumcision to minimize the possibility that participants' decisions would be influenced by the prospect of receiving a large net monetary gain.

This qualitative study enrolled a subsample of trial participants and their female partners. A stratified purposive sampling strategy was used to identify the qualitative study participants.^15^ Male participants were selected to be recruited according to whether or not they had undergone circumcision during the 2-month study period, their voucher amount, and location of residence. Approximately equal numbers of participants randomized to each of the 4 study groups were identified for recruitment. Individual in-depth interviews (IDIs) were conducted with participants after they either became circumcised during the RCT or chose not to during the eligibility period. Forty-five male participants, 19 of whom underwent circumcision during the trial and 26 of whom did not. IDIs were also conducted with 19 female partners of men enrolled in the trial, 10 with circumcised partners and 9 with uncircumcised partners. Research assistants (RAs) contacted selected participants by phone to request an interview; those not reachable after 3 attempts were considered ineligible. In order not to bias the sample of participants by income level, those who did not have a phone were visited in person by RAs. Those unavailable after 2 in-person attempts were considered ineligible. In order not to violate the confidentiality of their medical information, we sought permission from male participants with partners before contacting their female partners for an interview. Two participants refused to have their partners contacted and 2 women refused to participate. Women were required to know about the study and food voucher to participate; very few women were not aware of the study when contacted. Women were contacted either by phone or in person as described and male partners were not present during interviews.

Interviews were conducted by male and female RAs in either English or Dholuo, the primary local languages. All interviews were conducted using a semi-structured interview guide and in a private location. RAs also obtained consent from participants to audio-record interviews in addition to taking notes. Participants received refreshments valued at KES 100 (US $1.25) and compensation for transport if the interview was not conducted at the participant's home.

After each interview, RAs translated and transcribed interviews verbatim and then read several transcripts for quality and corrected errors; these were included in the analysis. Research team leaders provided comments to RAs on the initial round of transcripts to improve the quality of interviews. The study investigators created structural codes based on the interview guide and coded all transcripts. After coding the first few transcripts, the investigators compared application of codes and reconciled any discrepancies in the application of codes. The investigators conducted an inductive thematic analysis using a data-reduction table to identify patterns in participants' decision-making and the role played by the food voucher offers in these decisions.

## RESULTS

The majority (79%) of qualitative study participants who became circumcised and nearly all of those who did not (96%) were married (Table 1). The average number of dependents, both within and outside of the household, was 8.5 for circumcised participants and 7.6 for uncircumcised participants. More than half of both circumcised (53%) and uncircumcised (62%) participants were employed in occupations involving heavy manual labor (eg, farming and fishing). Approximately one quarter were employed in professions involving no manual labor (eg, teaching or office work), and the remainder were employed in professions involving some manual labor (eg, drivers, cooks). Overall, the characteristics of the qualitative participants were similar to those found in the parent study; however, it should be noted that this was not designed to be a representative sample. While the study attempted to recruit male participants for IDIs evenly across the 4 study groups, the distribution of participants across groups varied as a significantly higher proportion of participants receiving the $8.75 and $15.00 vouchers became circumcised in the RCT.^13^ The distribution of voucher amounts was more balanced among those who did not become circumcised.

**TABLE 1.**
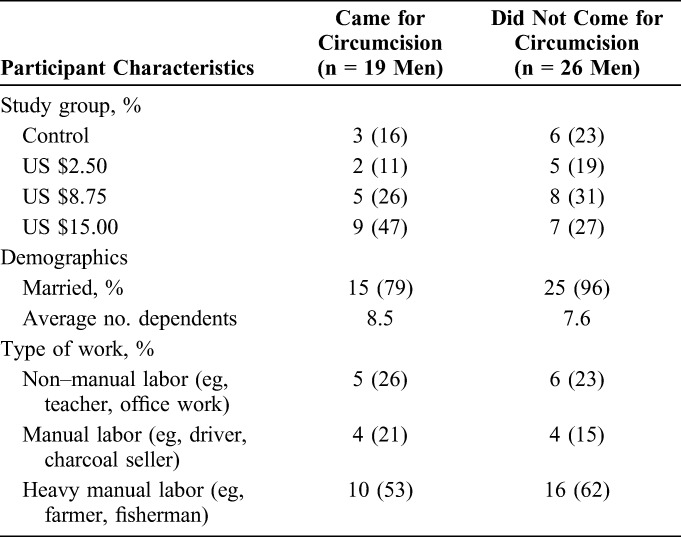
Male Participant Characteristics, by Circumcision Uptake Status

The majority of participants said that the financial costs of getting circumcised were a major barrier. Fear of pain was the second most commonly reported barrier. Participants were concerned with missing work and providing for their families during the postcircumcision healing period. A few mentioned the fear of losing their job while they were healing.

I had always wanted to go, but now I am the breadwinner. When I go, I will be forced to rest for a while and my family won't have food to eat, so it has made me hold back a bit.

—Fisherman, remained uncircumcised, US $15.00 group

…when you get circumcised, you can't find a way for fending for your family and when the school wants money from parents for their children, you can't do anything. The child gets chased from school and there is nothing the parent can do. The second thing is if you don't go to work and take some days off, when you go back, you will find another person has been filled in your space. It was a big problem.

—Farmer, became circumcised, US $8.75 group

For many participants who became circumcised during the study, the offer of a food voucher helped address their financial concerns and while most participants said the voucher did not cover all their lost wages and transportation costs, the amount was sufficient to influence them to go for VMMC.

Participants who became circumcised fell into 3 main categories; the first said that the offer of food vouchers was highly influential in their decision because it addressed their financial concerns. For this group, the offer of food vouchers removed the financial barrier to circumcision and these participants stated that they would not have sought circumcision without such compensation.

Looking at the duties that I do in a day, I earn (US $2.50) out of them. So that means that the (US $15) (voucher) was enough to cover roughly 6 days and for the other days, I would see what to do. So that is how the voucher changed my mind and influenced me.

—Casual laborer, became circumcised, US $15.00 group

A second group of participants said that they had already decided to get circumcised before enrolling in the study, but the offer of conditional economic compensation was a cue to action that motivated them and made it easier for them to go. They made statements like “*it helped me to some extent,*” *and* “*it eased my budget.*” Many of these participants did not seem to have a plan to go in the immediate future; they may have sought VMMC eventually, but the voucher facilitated the process.

The scratch card (food voucher) did not change my mind, though the amount helped me since I did not work for it. Circumcision is something I had always wanted. It made the decision as to whether to go for circumcision easier, and some of the things I had redeemed helped me for a good while.

—Cook, became circumcised, US $8.75 group

A final group of participants said they were motivated to get circumcised by conversations with study staff and information received during study enrollment.

(The) food voucher was just an added advantage. The person who gave me the food voucher is the one who influenced me given the way he spoke to me gently and told me a lot about circumcision.

—Clerical officer, became circumcised, US $15.00 group

The majority of participants who did not get circumcised had financial concerns associated with circumcision; half said this was their primary reason for not getting circumcised. A number of participants stated that the food voucher amount was inadequate to provide for their families during recovery. These reactions were found among uncircumcised participants in each of the study groups.

(US$15) is not enough for even 2 days, and somebody like me who…can even make more than (US $50) from morning to mid-day, now (US $15) cannot make me go for circumcision.

—Fisherman, remained uncircumcised, US $15.00 group

A number of participants said that while they were concerned about finances, they had another primary reason for not getting circumcised. While interested in getting circumcised, they indicated that they had not gone because they had not discussed circumcision with their female partners or because their partners had refused; they feared pain; or they felt the decision to get circumcised should not be linked to any sort of compensation.

There would not be any difference even if you are provided (me) with a million shillings, there would not be a difference. Yes, no one should pay you. You should just decide in your heart whether or not to go alone to protect own life.

—Artisan, remained uncircumcised, US $8.75 group

A couple of participants said events beyond their control such as illness or a death in the family prevented them from getting circumcised during the 2-month period. A few participants were not interested in getting circumcised because of religious or cultural reasons.

The vast majority of partners of both circumcised and uncircumcised participants were supportive of their partners getting circumcised and remained so after the trial. Their opinions of the compensation amounts varied, however. More than half reported that the compensation amounts were insufficient to sustain a family while recovery from the circumcision procedure took place.

I said to him depending on the work you want to do today, if you leave this place and use money going and coming back (to the clinic)… what is left is going to be little and what you are going to lose is much more. Instead do your work and leave the (voucher).

—Female partner of participant who remained uncircumcised, US $8.75 group

A small number of women said that the voucher amount was “just right”—one because “*it was something provided freely without having worked for it*” or had no opinion; none of the women reported that the amounts were too high. Women whose partners were in the control and US $2.50 groups expressed negative opinions of the compensation amounts, whereas women whose partners received US $8.75 or US $15.00 generally had more positive opinions of the economic intervention and felt that the compensation amounts were adequate.

Finally, there was no evidence that the provision of economic compensation was perceived as being coercive. When circumcised participants were asked about the compensation amounts, they were evenly split between saying it was the right amount or *too little*. Even in the $15 group, among those who said that the voucher was the right amount, most said that it did not cover all their lost wages from circumcision. Circumcised participants were specifically asked, “Was the voucher amount so high that you felt you could not turn it down?” No participants perceived the amount of compensation to be coercive, although 1 circumcised participant expressed feelings of obligation to participate following randomization.

## DISCUSSION

The results indicate that financial concerns, particularly the prospect of lost wages, continue to be an important consideration with regard to VMMC uptake for many adult men. This was true among participants who became circumcised during the study and those who did not. In addition, the results suggest that compensation in the form of food vouchers conditional on becoming circumcised was effective because they partially offset participants' opportunity costs associated with getting circumcised. For the majority of participants who chose not to get circumcised, the voucher amounts were perceived as being inadequate relative to their expected circumcision-related costs. Additionally, noneconomic barriers were identified by other uncircumcised men.

Interviews with participants who became circumcised provided support for both the hypothesized reasons why incentives or compensation provision are effective in influencing individual behaviors. Some participants indicated that they chose to undergo circumcision because the food vouchers partially offset circumcision-related economic costs. Other participations reported going for circumcision because the intervention reduced the immediate costs by a little bit and “nudged” them toward undertaking a decision that they had previously been intending to undertake in the near future. This latter result is consistent with the possibility that small incentives can help overcome decision-making biases identified in the behavioral economics literature, such as the tendency to place disproportionately greater weight on *immediate* costs and benefits.^11,14,16^

The results also underscore that in order for economic interventions to be highly effective, it is important that incentive or compensation amounts are chosen correctly. In interviews with participants who did not become circumcised, a common finding was that compensation amounts were too small given how much participants reported earning. Compensation amounts exceeding US $15.00 may therefore need to be offered to achieve higher VMMC uptake.

This study is noteworthy in light of growing interest in the use of economic incentives in the HIV prevention community. Although several studies have shown that economic incentives (including conditional cash transfer programs) can promote HIV testing uptake^17^ and reduce HIV prevalence among young women by increasing education,^18^ there have been relatively few attempts to assess specific reasons why incentives have or have not been effective in certain subpopulations. This study offers insights on specific barriers addressed by compensation provision, suggests ways in which the intervention can be made more effective, and reveals the need for entirely different noneconomic interventions such as increased information or education about circumcision from knowledgeable individuals.

The relatively small sample size in this study is among its key limitations. However, given the sampling strategy and the fact that the sample reflects the study population and outcomes of the study, the results provide important insight into the effect of economic interventions on VMMC uptake in this population. Circumcision demand creation efforts and services have been widely implemented in the Nyanza area over the past 5 years, and many men have already become circumcised. Study participants could therefore differ from other men in the region in that those remaining uncircumcised perceive greater barriers than those who have already gone for circumcision. It is also likely that our findings about the role of economic factors could be specific to Kenya; similar research on compensation provision should be undertaken elsewhere.

Despite these limitations, this study provides important insights into how economic interventions influence decision-making about VMMC. Men explain a detailed thought process by which they weighed the costs and benefits of becoming circumcised and assessed whether the voucher was sufficient to outweigh the costs incurred through loss of income and transportation. Although the vouchers offered were not effective for addressing all men's concerns with circumcision, they have been shown to be an important tool for increasing circumcision uptake among some adult men and warrant further consideration in future VMMC demand creation efforts.
